# Behavioral Graded Activity^+^ (BGA^+^) for Osteoarthritis: A Paradigm Shift from Disease-Based Treatment to Personalized Activity Self-Management

**DOI:** 10.3390/jcm9061793

**Published:** 2020-06-09

**Authors:** Jo Nijs, Kelly Ickmans, David Beckwée, Laurence Leysen

**Affiliations:** 1 Pain in Motion International Research Group, Department of Physiotherapy, Human Physiology and Anatomy, Faculty of Physical Education & Physiotherapy, Vrije Universiteit Brussel, BE-1090 Brussels, Belgium; kelly.ickmans@vub.be (K.I.); Laurence.Leysen@vub.be (L.L.); 2 Chronic Pain Rehabilitation, Department of Physical Medicine and Physiotherapy, University Hospital Brussels, BE1090 Brussels, Belgium; 3 Institute of Neuroscience and Physiology, University of Gothenburg, Box 430, SE-405 30 Göteborg, Sweden; 4 Flemish Research Foundation (FWO), BE1050 Brussels, Belgium; 5 Rehabilitation Research Group, Department of Physiotherapy, Human Physiology and Anatomy, Faculty of Physical Education & Physiotherapy, Vrije Universiteit Brussel, BE-1090 Brussels, Belgium; david.beckwee@vub.be

**Keywords:** rehabilitation, chronic pain, inflammation

## Abstract

Three promising directions for improving care for osteoarthritis (OA) include novel education strategies to target unhelpful illness and treatment beliefs; methods to enhance the efficacy of exercise interventions; and innovative, brain-directed treatments. Here we explain that each of those three promising directions can be combined through a paradigm-shift from disease-based treatments to personalized activity self-management for patients with OA. Behavioral graded activity (BGA) accounts for the current understanding of OA and OA pain and allows a paradigm shift from a disease-based treatment to personalized activity self-management for patients with OA. To account for the implementation barriers of BGA, we propose adding pain neuroscience education to BGA (referred to as BGA^+^). Rather than focusing on the biomedical (and biomechanical) disease characteristics of OA, pain neuroscience education implies teaching people about the underlying biopsychosocial mechanisms of pain. To account for the lack of studies showing that BGA is “safe” with respect to disease activity and the inflammatory nature of OA patients, a trial exploring the effects of BGA^+^ on the markers of inflammation is needed. Such a trial could clear the path for the required paradigm shift in the management of OA (pain) and would allow workforce capacity building that de-emphasizes biomedical management for OA.

## 1. Introduction

Given the high number of treatment guidelines available, clinicians might be overwhelmed by the evidence on osteoarthritis (OA) management. Taking into account the socio-economic impact and high prevalence of OA, it is imperative that healthcare professionals have free access to up-to-date, evidence-based information to assist them in treatment decision-making. Therefore, in the *Journal of Clinical Medicine*‘s Special Issue on “Rehabilitation for Persistent Pain Across the Lifespan”, Rice et al. provide a state-of-the-art review of rehabilitation for OA pain [1]. In addition to providing a comprehensive and easily consumable overview of the best evidence on rehabilitation for OA pain, they also explore promising directions for clinical practice and discuss potential future research avenues. The promising directions for clinical practice include novel education strategies to target unhelpful illness and treatment beliefs; methods to enhance the efficacy of exercise interventions; and innovative, brain-directed treatments [1]. Here we explain that each of those three promising directions can be combined through a paradigm-shift from disease-based treatments to personalized activity self-management for patients with OA. 

## 2. Disease-Based Osteoarthritis Treatment Offers Modest Effect Sizes

Current OA treatment guidelines have a disease-based, biomedical focus (e.g., joint replacement surgery). Even education and exercise therapy, cornerstones of international OA treatment guidelines [2,3,4,5], have a disease-based focus; education typically consists of accurate information to enhance the understanding of the condition (hip or knee OA) and its consequences, while the progression of exercises is typically guided by tolerable levels of pain and the patient’s ability to perform a given exercise. Such a disease-based, biomedical focus is offline with our current understanding of OA pain [6], including its biopsychosocial nature and the role of central sensitization in amplifying the OA pain experience [7]. The effect sizes of disease-based education and exercise therapy for OA are moderate at best [5], allowing room for improvement. Such improvement might come from modifying existing disease-based educational and exercise therapy interventions to our current understanding of OA pain [6,8].

## 3. Personalized Activity Self-Management for Patients with Osteoarthritis

High all-cause mortality from knee OA is mediated mainly through walking disability [9], and encouraging people to walk and “get out and about” in addition to targeting OA can be protective against excessive mortality [10]. However, to people with OA, pain represents a major barrier to walking or any other physical activity [11,12,13]. An innovative and effective way of accounting for the current biopsychosocial understanding of OA pain and encouraging people to walk and “get out and about” and addressing pain as a barrier to physical activity, is through using behavioral-graded activity (BGA). BGA is a behavioral treatment integrating the concept of operant conditioning to increase the level of physical activity in the patient’s daily life [14]. It is a highly personalized approach, targeting the patients’ activity limitations and self-defined treatment goals (goal setting), and individually tailoring the baseline and grading levels for performing these daily activities. BGA allows OA patients to shift from having priority in pain control to priority in valued life goals.

The use of BGA for patients with OA pain is supported by a cluster-randomized clinical trial [14,15], suggesting that BGA is an effective treatment for relieving pain, improving physical functioning and physical performance, and preventing joint replacement surgery in patients with OA [14,15]. Compared to usual care, BGA resulted in superior exercise adherence and more physical activity in people with hip or knee OA [16]. Such increased physical activity levels not only result in less pain [17] but also hold the potential to decrease the low-grade inflammation that is characteristic of OA. Indeed, OA is characterized by a chronic low-grade inflammatory profile [18,19,20], and physical activity has strong anti-inflammatory effects [21,22,23,24,25,26], but studies in OA remain scarce. With its anti-inflammatory action, physical activity might even contribute to decreasing the mortality risk in patients with OA [27,28,29].

## 4. Implementation Barriers for a Personalized Activity Self-Management Approach

BGA accounts for the current understanding of OA and OA pain and allows a paradigm shift from a disease-based treatment to personalized activity self-management for patients with OA (Table 1). However, more than 10 years after the initial trial findings were published, BGA is not recommended by the European [30], American [4], or international [3] guidelines for OA management, preventing its implementation in clinical practice. Another implementation barrier relates to the common belief among both patients and therapists that OA pain is a “warning sign” of disease severity. Up to 89% of future therapists consider severe pain a reason for not using exercise in the treatment of OA, while 87% believe that increasing overall activity levels cannot stop the knee problem getting worse [31]. A multinational study concluded that workforce capacity building that de-emphasizes biomedical management for OA is urgently needed [32]. Beliefs about the consequences of exercise account for general practitioners’ use of exercise in knee pain [33] (with 29% believing that rest was the optimum management approach for knee OA [34]). To account for these implementation barriers, we propose adding pain neuroscience education to BGA (referred to as BGA^+^).

## 5. Pain Neuroscience Education + Behavioral Graded Activity = BGA^+^

Indeed, patients with OA are confused about the cause of their pain and bewildered by its variability and randomness [35]. Without adequate information and advice from healthcare professionals, people do not know what they should and should not do, and, as a consequence, avoid activity for fear of causing harm. A Cochrane review concluded that providing reassurance and clear advice about the value of exercise in OA and opportunities to participate in exercise programs that people regard as enjoyable and relevant to their personal life may encourage greater exercise participation, which brings a range of health benefits to patients with OA [36]. Therefore, the addition of pain neuroscience education to BGA entails a dramatic shift in educating OA patients prior to exercise/activity interventions.

As explained by Rice et al. [1], rather than focusing on the biomedical (and biomechanical) disease characteristics of OA, pain neuroscience education implies teaching people about the underlying biopsychosocial mechanisms of pain. Pain neuroscience education is a remarkable example of how patients who receive proper guidance can really help themselves. Pain neuroscience education prepares patients for a time-contingent (“Perform the activity/exercise for five minutes, regardless of the pain.”), cognition-targeted approach to daily (physical) activity and exercise therapy, as typically applied during BGA. Such a time-contingent approach replaces the classical symptom-contingent (“Stop the activity/exercise once it hurts”) approach. A time-contingent approach to activities is often difficult for patients to comprehend and comply with and for therapists to implement. Indeed, a time-contingent approach to activities/exercises contradicts with the disease-based, biomedical focus on exercise and activity interventions with which therapists are often more comfortable with [37]. Pain neuroscience education specifically addresses this barrier for making a the paradigm shift to personalized activity self-management for patients with OA, both at the patient and the therapist level.

In a recent proof-of-concept study, we reported that pain neuroscience education, compared to biomedical-focused education, generates favorable effects on decreasing pain catastrophizing, excessive attention to pain, and activity-related fear at short and long-term follow-ups in people with knee OA [38]. In addition, a case series of 12 OA patients receiving pain neuroscience education prior to joint replacement surgery revealed that the fear of movement and sensitivity to pain decreased [39].

## 6. Conclusions

Taken together, BGA^+^ addresses all three promising directions for clinical practice highlighted by Rice et al. [1]. BGA^+^ includes a novel education strategy to target unhelpful illness and treatment beliefs; applies evidence-based methods to enhance the efficacy of physical activity/exercise interventions; and can be considered an innovative, brain-directed treatment. Hence, BGA^+^ allows a paradigm-shift from disease-based treatments to personalized activity self-management for patients with OA. Still, to account for the implementation barrier, where OA patients fear that moving despite pain might aggravate disease activity, as well as the lack of studies showing that BGA is “safe” with respect to disease activity and the inflammatory nature of OA patients (therapist barrier), a trial exploring the effects of BGA^+^ on the markers of inflammation in patients with OA is urgently needed. Such a trial could clear the path for the required paradigm shift in the management of OA (pain) and would allow workforce capacity building that de-emphasizes biomedical management for OA [32].

## Figures and Tables

**Table 1:** Proposed paradigm shift from disease-based treatment to personalized activity self-management for osteoarthritis.

Current Best-Evidence: Disease-Based Treatment	Personalized Activity Self-Management
Disease-based, biomedically focussed eduation	Biopsychosocial education
Exercises target muscle strength, endurance, motor control, etc.	Physical activities targeting self-defined functional and/or social activities and life goals
Pain = sign of tissue damage, including inflammation	Pain = sign of nervous system sensitivity
Pain contingent approach to grading exercises	Operant conditioning and time contingent approach to grading daily activities

## References

[1] Rice D., McNair P., Huysmans E., Letzen J., Finan P. (2019). Best Evidence Rehabilitation for Chronic Pain Part 5: Osteoarthritis. J. Clin. Med..

[2] Zhang W., Moskowitz R.W., Nuki G., Abramson S., Altman R.D., Arden N., Bierma-Zeinstra S., Brandt K.D., Croft P., Doherty M. (2008). OARSI recommendations for the management of hip and knee osteoarthritis, Part II: OARSI evidence-based, expert consensus guidelines. Osteoarthr. Cartil..

[3] McAlindon T.E., Bannuru R.R., Sullivan M.C., Arden N.K., Berenbaum F., Bierma-Zeinstra S.M., Hawker G.A., Henrotin Y., Hunter D.J., Kawaguchi H. (2014). OARSI guidelines for the non-surgical management of knee osteoarthritis. Osteoarthr. Cartil..

[4] Hochberg M.C., Altman R.D., April K.T., Benkhalti M., Guyatt G., McGowan J., Towheed T., Welch V., Wells G., Tugwell P. (2012). American College of Rheumatology 2012 recommendations for the use of nonpharmacologic and pharmacologic therapies in osteoarthritis of the hand, hip, and knee. Arthritis Care Res..

[5] Fransen M., McConnell S., Harmer A.R., Van der Esch M., Simic M., Bennell K.L. (2015). Exercise for osteoarthritis of the knee. Cochrane Database Syst. Rev..

[6] Lluch Girbes E., Meeus M., Baert I., Nijs J. (2014). Balancing “hands-on” with “hands-off” physical therapy interventions for the treatment of central sensitization pain in osteoarthritis. Man. Ther..

[7] Lluch E., Torres R., Nijs J., Van Oosterwijck J. (2014). Evidence for central sensitization in patients with osteoarthritis pain: A systematic literature review. Eur. J. Pain.

[8] Lluch Girbes E., Nijs J., Torres-Cueco R., Lopez C. (2013). Pain treatment for patients with osteoarthritis and central sensitization. Phys. Ther..

[9] Liu Q., Niu J., Li H., Ke Y., Li R., Zhang Y., Lin J. (2017). Knee Symptomatic Osteoarthritis, Walking Disability, NSAIDs Use and All-cause Mortality: Population-based Wuchuan Osteoarthritis Study. Sci. Rep..

[10] Wilkie R., Parmar S.S., Blagojevic-Bucknall M., Smith D., Thomas M.J., Seale B.J., Mansell G., Peat G. (2019). Reasons why osteoarthritis predicts mortality: Path analysis within a Cox proportional hazards model. RMD Open.

[11] Dobson F., Hinman R.S., Roos E.M., Abbott J.H., Stratford P., Davis A.M., Buchbinder R., Snyder-Mackler L., Henrotin Y., Thumboo J. (2013). OARSI recommended performance-based tests to assess physical function in people diagnosed with hip or knee osteoarthritis. Osteoarthr. Cartil..

[12] Davis M.A., Ettinger W.H., Neuhaus J.M., Mallon K.P. (1991). Knee osteoarthritis and physical functioning: Evidence from the NHANES I Epidemiologic Followup Study. J. Rheumatol..

[13] Collins J.E., Katz J.N., Dervan E.E., Losina E. (2014). Trajectories and risk profiles of pain in persons with radiographic, symptomatic knee osteoarthritis: Data from the osteoarthritis initiative. Osteoarthr. Cartil..

[14] Veenhof C., Koke A.J., Dekker J., Oostendorp R.A., Bijlsma J.W., van Tulder M.W., van den Ende C.H. (2006). Effectiveness of behavioral graded activity in patients with osteoarthritis of the hip and/or knee: A randomized clinical trial. Arthritis Rheum..

[15] Pisters M.F., Veenhof C., Schellevis F.G., De Bakker D.H., Dekker J. (2010). Long-term effectiveness of exercise therapy in patients with osteoarthritis of the hip or knee: A randomized controlled trial comparing two different physical therapy interventions. Osteoarthr. Cartil..

[16] Pisters M.F., Veenhof C., de Bakker D.H., Schellevis F.G., Dekker J. (2010). Behavioural graded activity results in better exercise adherence and more physical activity than usual care in people with osteoarthritis: A cluster-randomised trial. J. Physiother..

[17] van Baar M.E., Assendelft W.J., Dekker J., Oostendorp R.A., Bijlsma J.W. (1999). Effectiveness of exercise therapy in patients with osteoarthritis of the hip or knee: A systematic review of randomized clinical trials. Arthritis Rheum..

[18] Daghestani H.N., Kraus V.B. (2015). Inflammatory biomarkers in osteoarthritis. Osteoarthr. Cartil..

[19] Greene M.A., Loeser R.F. (2015). Aging-related inflammation in osteoarthritis. Osteoarthr. Cartil..

[20] Runhaar J., Bierma-Zeinstra S.M.A. (2017). *S*hould exercise therapy for chronic musculoskeletal conditions focus on the anti-inflammatory effects of exercise?. Br. J. Sports Med..

[21] Petersen A.M., Pedersen B.K. (2005). The anti-inflammatory effect of exercise. J. Appl. Physiol..

[22] Forti L.N., Van Roie E., Njemini R., Coudyzer W., Beyer I., Delecluse C., Bautmans I. (2017). Effects of resistance training at different loads on inflammatory markers in young adults. Eur. J. Appl. Physiol..

[23] Liberman K., Forti L.N., Beyer I., Bautmans I. (2017). The effects of exercise on muscle strength, body composition, physical functioning and the inflammatory profile of older adults: A systematic review. Curr. Opin. Clin. Nutr. Metab. Care.

[24] Bautmans I., Njemini R., Vasseur S., Chabert H., Moens L., Demanet C., Mets T. (2005). Biochemical changes in response to intensive resistance exercise training in the elderly. Gerontology.

[25] Forti L.N., Van Roie E., Njemini R., Coudyzer W., Beyer I., Delecluse C., Bautmans I. (2016). Load-Specific Inflammation Mediating Effects of Resistance Training in Older Persons. J. Am. Med. Dir. Assoc..

[26] Beyer I., Mets T., Bautmans I. (2012). Chronic low-grade inflammation and age-related sarcopenia. Curr. Opin. Clin. Nutr. Metab. Care.

[27] Wang Y., Nguyen U.D.T., Lane N.E., Lu N., Wei J., Lei G., Zeng C., Zhang Y. (2020). Knee osteoarthritis, potential mediators, and risk of all-cause mortality: Data from the Osteoarthritis Initiative. Arthritis Care Res..

[28] Hawker G.A., Croxford R., Bierman A.S., Harvey P.J., Ravi B., Stanaitis I., Lipscombe L.L. (2014). All-cause mortality and serious cardiovascular events in people with hip and knee osteoarthritis: A population based cohort study. PLoS ONE.

[29] Veronese N., Cereda E., Maggi S., Luchini C., Solmi M., Smith T., Denkinger M., Hurley M., Thompson T., Manzato E. (2016). Osteoarthritis and mortality: A prospective cohort study and systematic review with meta-analysis. Semin. Arthritis Rheum..

[30] Fernandes L., Hagen K.B., Bijlsma J.W., Andreassen O., Christensen P., Conaghan P.G., Doherty M., Geenen R., Hammond A., Kjeken I. (2013). EULAR recommendations for the non-pharmacological core management of hip and knee osteoarthritis. Ann. Rheum. Dis..

[31] Vertommen B. (2014). Pain Attitudes and Beliefs of Physiotherapy Students Concerning the Management of Osteoarthritis.

[32] Briggs A.M., Hinman R.S., Darlow B., Bennell K.L., Leech M., Pizzari T., Greig A.M., MacKay C., Bendrups A., Larmer P.J. (2019). *C*onfidence and Attitudes Toward Osteoarthritis Care Among the Current and Emerging Health Workforce: A Multinational Interprofessional Study. ACR Open Rheumatol..

[33] Cottrell E., Roddy E., Rathod T., Porcheret M., Foster N.E. (2016). What influences general practitioners’ use of exercise for patients with chronic knee pain? Results from a national survey. BMC Fam. Pract..

[34] Cottrell E., Roddy E., Foster N.E. (2010). The attitudes, beliefs and behaviours of GPs regarding exercise for chronic knee pain: A systematic review. BMC Fam. Pract..

[35] Selten E.M.H., Geenen R., Schers H.J., van den Hoogen F.H.J., van der Meulen-Dilling R.G., van der Laan W.H., Nijhof M.W., van den Ende C.H.M., Vriezekolk J.E. (2018). Treatment Beliefs Underlying Intended Treatment Choices in Knee and Hip Osteoarthritis. Int. J. Behav. Med..

[36] Hurley M., Dickson K., Hallett R., Grant R., Hauari H., Walsh N., Stansfield C., Oliver S. (2018). Exercise interventions and patient beliefs for people with hip, knee or hip and knee osteoarthritis: A mixed methods review. Cochrane Database Syst. Rev..

[37] Nijs J., Roussel N., Paul van Wilgen C., Koke A., Smeets R. (2013). Thinking beyond muscles and joints: Therapists’ and patients’attitudes and beliefs regarding chronic musculoskeletal pain are key to applying effective treatment. Man. Ther..

[38] Lluch E., Duenas L., Falla D., Baert I., Meeus M., Sanchez-Frutos J., Nijs J. (2018). Preoperative Pain Neuroscience Education Combined With Knee Joint Mobilization for Knee Osteoarthritis: A Randomized Controlled Trial. Clin. J. Pain..

[39] Louw A., Zimney K., Reed J., Landers M., Puentedura E. (2019). Immediate preoperative outcomes of pain neuroscience education for patients undergoing total knee arthroplasty: A case series. Physiother. Theory Pract..

